# Association between tri-ponderal mass index and glucose metabolism disorder in children with obesity in China: A case–control study

**DOI:** 10.1097/MD.0000000000037364

**Published:** 2024-03-08

**Authors:** Dongguang Zhang, Yu Yang, Lei Xu, Haiying Zou, Xian Wu, Li Yang, Bin Zhou, Qingbo Xu

**Affiliations:** a Department of Endocrinology, Metabolism and Genetics, Jiangxi Provincial Children’s Hospital (The Affiliated Children’s Hospital of Nanchang Medical College), Jiangxi Provincial Children’s Genetic and Metabolic Disease Clinical Medicine Research Center, Nanchang, China.

**Keywords:** adolescent, child, glucose metabolism disorder, obesity, tri-ponderal mass index

## Abstract

Obesity is a risk factor for glucose metabolism disorder. This study explored the association between the tri-ponderal mass index (TMI) and indicators of glucose metabolism disorder in children with obesity in China. This retrospective case–control study included children aged 3 to 18 years old diagnosed with obesity at Jiangxi Provincial Children’s Hospital (China) between January 2020 and April 2022. Demographic and clinical characteristics were obtained from the medical records. Factors associated with glucose metabolism disorder were explored by logistic regression analysis. Pearson correlations were calculated to evaluate the relationships between TMI and indicators of glucose metabolism disorder. The analysis included 781 children. The prevalence of glucose metabolism disorder was 22.0% (172/781). The glucose metabolism disorder group had an older age (11.13 ± 2.19 vs 10.45 ± 2.33 years old, *P* = .001), comprised more females (76.8% vs 66.9%, *P* = .008), had a higher Tanner index (*P* = .001), and had a larger waist circumference (89.00 [82.00–95.00] vs 86.00 [79.00–93.75] cm, *P* = .025) than the non-glucose metabolism disorder group. There were no significant differences between the glucose metabolism disorder and non-glucose metabolism disorder groups in other clinical parameters, including body mass index (26.99 [24.71–30.58] vs 26.57 [24.55–29.41] kg/m^2^) and TMI (18.38 [17.11–19.88] vs 18.37 [17.11–19.88] kg/m^3^). Multivariable logistic regression did not identify any factors associated with glucose metabolism disorder. Furthermore, TMI was only very weakly or negligibly correlated with indicators related to glucose metabolism disorder. TMI may not be a useful indicator to screen for glucose metabolism disorder in children with obesity in China.

## 1. Introduction

Childhood obesity is now considered a global health problem.^[1,2]^ The prevalence of overweight and obesity among Chinese children aged 7 to 18 years old increased 55-fold from 1985 to 2014 and around 1.5-fold from 2010 to 2014.^[3]^ The National Nutrition Survey in China reported that the prevalence of overweight and obesity increased from 5.3% in 1995 to 20.5% in 2014,^[4]^ and a more recent study found that the prevalence of overweight or obesity in children aged 6 to 17 years old in Suzhou was 27.8%.^[5]^ Children with obesity are at increased risk of developing chronic diseases such as glucose metabolism disorder,^[6]^ hypertension,^[7,8]^ nonalcoholic fatty liver disease (NAFLD),^[9]^ and psychological disorders.^[10]^ Furthermore, childhood obesity is associated with premature death in adulthood.^[11]^

Type 2 diabetes mellitus (T2DM) has become increasingly common in children in recent years,^[12]^ and the prevalence of pediatric T2DM in China is as high as 15 per 100,000 children in some regions.^[13]^ Obesity is recognized as an important risk factor for T2DM in children.^[6,14]^ Specific risk factors for T2DM in schoolchildren with overweight/obesity include family history of T2DM, maternal diabetes/gestational diabetes, and hypertension.^[15]^ T2DM in childhood is associated with the development of diabetic complications (e.g., hypertension, retinopathy, and nephropathy) and cardiovascular disease.^[16]^ The timely diagnosis of pediatric T2DM is essential to allow the implementation of management strategies such as lifestyle modifications and oral hypoglycemic agents to reduce insulin requirements and inhibit the development of complications. A formal diagnosis of T2DM and insulin resistance is based on fasting and random plasma glucose levels, oral glucose tolerance tests (OGGTs), and glycated hemoglobin (HbA1c) levels.^[16]^ However, these tests require blood samples and laboratory analyses. The identification of a simple marker of glucose metabolism disorder in children with obesity would allow the formal diagnostic tests to be targeted to those at a higher risk of insulin resistance and T2DM.

Body mass index (BMI) is the most widely used indicator of adiposity in children, with cutoffs for overweight and obesity based on age and sex percentiles.^[17]^ Still, it is recognized that BMI has limited ability to differentiate between increased fat mass and increased muscle mass as the cause of excess weight.^[17]^ The tri-ponderal mass index (TMI) is an alternative indicator of adiposity that is calculated as weight (kg)/height cubed (m^3^). There is evidence that TMI is more accurate than BMI at estimating body fat levels in non-Hispanic white children aged 8 to 17 years old.^[18]^ Furthermore, a recent study concluded that TMI was superior to BMI and BMI *z*-score in the prediction of central obesity and hypertension in adolescents with overweight.^[19]^ Interestingly, TMI was reported to be a better predictor of metabolic syndrome than BMI in adolescents aged 11 to 19 years old in Iran.^[20]^ Furthermore, TMI was found to have moderate discriminatory ability in the detection of metabolic syndrome in children and adolescents in Colombia.^[21]^ TMI was shown to have a good ability to detect metabolic syndrome in children aged 7 to 18 years old in China and children aged 12 to 18 years old in the USA.^[22]^

However, there are limited data regarding the ability of TMI to diagnose T2DM in children with obesity. Therefore, this study aimed to explore the association between TMI and indicators of glucose metabolism disorder in children with obesity in China.

## 2. Materials and methods

### 2.1. Study design and patients

This retrospective case-control study included children diagnosed with obesity at the Department of Endocrinology, Genetics, and Metabolism, Jiangxi Provincial Children’s Hospital, Jiangxi, China, between January 2020 and April 2022. The inclusion criteria were (1) aged ≥3 years old and <18 years old, and (2) diagnosed with obesity according to the guidelines of the National Health and Family Planning Commission of the People’s Republic of China^[23]^ (Table 1). The exclusion criteria were (1) severe or chronic diseases, including cancer, (2) previous central nervous system surgery, (3) history of drug therapy (e.g., with glucocorticoids) that could induce weight gain, and (4) acute infection, trauma, or other stressors (to avoid the potential influence of stress-induced blood glucose elevation). This study was approved by the Ethics Committee of Jiangxi Provincial Children’s Hospital. The requirement for informed consent was waived as this was a retrospective analysis of anonymized data.

**Table 1:** Screening for overweight and obesity among school-age children and adolescents.

Age (year)	Boy		Girl	
	Overweight	Obesity	Overweight	Obesity
6.0~	16.4	17.7	16.2	17.5
6.5~	16.7	18.1	16.5	18
7.0~	17	18.7	16.8	18.5
7.5~	17.4	19.2	17.2	19
8.0~	17.8	19.7	17.6	19.4
8.5~	18.1	20.3	18.1	19.9
9.0~	18.5	20.8	18.5	20.4
9.5~	18.9	21.4	19	21
10.0~	19.2	21.9	19.5	21.5
10.5~	19.6	22.5	20	22.1
11.0~	19.9	23	20.5	22.7
11.5~	20.3	23.6	21.1	23.3
12.0~	20.7	24.1	21.5	23.9
12.5~	21	24.7	21.9	24.5
13.0~	21.4	25.2	22.2	25
13.5~	21.9	25.7	22.6	25.6
14.0~	22.3	26.1	22.8	25.9
14.5~	22.6	26.4	23	26.3
15.0~	22.9	26.6	23.2	26.6
15.5~	23.1	26.9	23.4	26.9
16.0~	23.3	27.1	23.6	27.1
16.5~	23.5	27.4	23.7	27.4
17.0~	23.7	27.6	23.8	27.6
17.5~	23.8	27.8	23.9	27.8
18.0~	24	28	24	28

### 2.2. Data collection and definitions

The following baseline demographic and clinical data were obtained from the medical records: age, sex, BMI, TMI, Tanner stage (sexual maturity rating),^[24]^ family history of diabetes mellitus, waist circumference, skin manifestations associated with obesity or T2DM (acanthosis nigricans or purple striae), systolic blood pressure, diastolic blood pressure, presence/absence of NAFLD, fasting blood glucose level (FBG), fasting blood insulin level (FBI), Homeostatic Model Assessment for Insulin Resistance value (HOMA-IR), HbA1c levels, 2-hour blood glucose and insulin levels during an oral glucose tolerance test (OGTT), and levels of alanine transaminase, free fatty acids, total cholesterol, triglycerides, high-density lipoprotein cholesterol and low-density lipoprotein cholesterol. The diagnosis of NAFLD was made if there was imaging or histologic evidence of hepatic steatosis in the absence of significant alcohol intake, and secondary causes had been excluded.^[9]^

TMI was calculated as body weight/height cubed (kg/m^3^).^[18]^ Indicators of glucose metabolism disorder included FBG, FBI, HOMA-IR, HbA1c, and OGTT results for 2-hour blood glucose and insulin levels. T2DM, impaired fasting glucose (IFG; a prediabetic condition) and impaired glucose tolerance (IGT; a prediabetic condition) were diagnosed in accordance with the Chinese Expert Consensus on Diagnosis and Treatment of Type 2 Diabetes in Children and Adolescents^[25]^: FBG ≥ 7.0 mmol/L or OGTT 2-hour blood glucose ≥ 11.1 mmol/L was used to diagnose T2DM; FBG of 5.6 to 6.9 mmol/L was taken to indicate IFG; and OGTT 2-hour blood glucose of 7.8 to 11.1 mmol/L was taken to indicate IGT. HOMA-IR was calculated as: [FBG (mmol/L) × FBI (μU/mL)]/22.5.^[26]^ Patients with HOMA-IR > 3.0 were considered to have insulin resistance, based on a previous report that the 95th percentile for HOMA-IR was 3.0 in healthy Chinese children and adolescents aged 6 to 18 years old.^[27]^

### 2.3. Statistical analysis

SPSS 26.0 (IBM Corp., Armonk, NY) was used for analysis. The participants were divided into the glucose metabolism disorder group and the non-glucose metabolism disorder group. Quantitative data were tested for normality. Normally distributed data were described as means ± standard deviations and compared between groups using the t-test for independent samples. Non-normally distributed parameters were presented as medians (interquartile ranges) and compared between groups using the Mann–Whitney U test. Qualitative data were described as frequency (constituent ratio or percentage) and analyzed using the chi-squared test. Univariable and multivariable logistic regression analyses were used to identify the factors associated with glucose metabolism disorder. Factors with *P* < .05 in the univariable analyses were entered into the multivariable analysis, and odds ratios and 95% confidence intervals were calculated. Pearson correlations were obtained to evaluate the relationships between TMI and indicators of glucose metabolism disorder. *P* < .05 was considered statistically significant.

## 3. Results

### 3.1. Baseline demographic and clinical characteristics of the study participants

Among the 781 study participants included in the final analysis, 172 children (22.0%) were diagnosed with glucose metabolism disorder, and 609 children (78.0%) did not have glucose metabolism disorder. Among the 172 children with glucose metabolism disorders, 28 were diagnosed with IFG, 130 were with IGT, and 14 with T2DM. The baseline demographic and clinical characteristics of the patients are shown in Table 2. The glucose metabolism disorder group had a significantly older age (11.13 ± 2.19 vs 10.45 ± 2.33 years old, *P* = .001), comprised significantly more females (76.8% vs 66.9%, *P* = .008), had a significantly higher Tanner index (*P* = .001), and had a significantly larger waist circumference (89.00 [82.00–95.00] vs 86.00 [79.00–93.75] cm, *P* = .025) than the non-glucose metabolism disorder group. Furthermore, the glucose metabolism disorder group had significantly higher FBG (4.97 [4.54–5.51] vs 4.69 [4.35–5.00] mmol/L, *P* < .001), FBI (21.47 [15.22–34.10] vs 18.60 [13.04–26.51] µIU/mL, *P* < .001), HOMA-IR (4.87 [3.43–7.60] vs 3.84 [2.66–5.50], *P* < .001), HbA1c (5.20% [4.80–5.60%] vs 5.03% [4.68–5.49%], *P* = .001), OGTT 2-hour blood glucose level (8.30 [7.91–9.15] vs 6.60 [6.03–7.14] mmol/L, *P* < .001) and OGTT 2-hour blood insulin level (165.45 [20.61–333.90] vs 89.48 [7.03–382.4] µIU/mL, *P* < .001). However, there were no significant differences between the glucose metabolism disorder and non-glucose metabolism disorder groups in BMI (26.99 [24.71–30.58] vs 26.57 [24.55–29.41] kg/m^2^) or TMI (18.38 [17.11–19.88] vs 18.37 [17.11–19.88] kg/m^3^). Additionally, there were no significant differences between the 2 groups in the family history of diabetes mellitus, the prevalence of acanthosis nigricans or purple striae in the skin, systolic blood pressure, diastolic blood pressure, the prevalence of NAFLD, or the levels of alanine transaminase, free fatty acids, total cholesterol, triglycerides, low-density lipoprotein cholesterol or high-density lipoprotein cholesterol (Table 2).

**Table 2:** Baseline characteristics of the study participants.

Characteristic	Non-glucose metabolism disorder group (n = 609)	Glucose metabolism disorder group (n = 172)	*P*
Age (years)	10.45 ± 2.33	11.13 ± 2.19	.001
Sex			.008
Male	468 (76.8%)	115 (66.9%)	
Female	141 (23.2%)	57 (33.1%)	
BMI (kg/m^2^)	26.57 (24.55–29.41)	26.99 (24.71–30.58)	.115
Family history of diabetes	95 (15.6%)	25 (14.5%)	.732
Tanner stage			.001
I	314 (51.6%)	63 (36.6%)	
II	121 (19.9%)	34 (19.8%)	
III	97 (15.9%)	36 (20.9%)	
IV	59 (9.7%)	25 (14.5%)	
V	18 (3.0%)	14 (8.1%)	
TMI (kg/m^3^)	18.37 (17.11–19.88)	18.38 (17.11–19.88)	.840
FBG (mmol/L)	4.69 (4.35–5.00)	4.97 (4.54–5.51)	<.001
FBI (µIU/mL)	18.60 (13.04–26.51)	21.47 (15.22–34.10)	<.001
HOMA-IR	3.84 (2.66–5.50)	4.87 (3.43–7.60)	<.001
HbA1c (%)	5.03 (4.68–5.49)	5.20 (4.80–5.60)	.001
OGTT 2-h glucose (mmol/L)	6.60 (6.03–7.14)	8.30 (7.91–9.15)	<.001
OGTT 2-h insulin (µIU/mL)	89.48 (7.03–382.4)	165.45 (20.61–333.90)	<.001
Acanthosis nigricans	335 (55.0%)	91 (52.9%)	.625
Purple striae of skin	227 (37.3%)	73 (42.4%)	.219
Waist circumference (cm)	86.00 (79.00–93.75)	89.00 (82.00–95.00)	.025
SBP (mm Hg)	110.00 (102.00–118.00)	110.00 (102.00–12.00)	.168
DBP (mm Hg)	66.00 (60.00–70.00)	65.00 (60.00–7.00)	.803
Fatty liver	281 (46.1%)	90 (52.3%)	.152
Alanine transaminase (U/L)	29.00 (21.00–50.00)	29.00 (21.00–58.23)	.608
Free fatty acids (mmol/L)	0.68 (0.50–0.86)	0.69 (0.50–0.85)	.953
Total cholesterol (mmol/L)	4.10 (3.71–4.60)	4.23 (3.81–4.74)	.066
Triglycerides (mmol/L)	1.07 (0.81–1.46)	1.07 (0.82–1.66)	.299
LDL-C (mmol/L)	2.42 (1.99–2.81)	2.46 (2.10–2.92)	.322
HDL-C (mmol/L)	1.16 (1.03–1.32)	1.18 (1.03–1.48)	.836

BMI = body mass index, DBP = diastolic blood pressure, FBG = fasting blood glucose, FBI = fasting blood insulin, HbA1c = glycated hemoglobin, HDL-C = high-density lipoprotein cholesterol, HOMA-IR = Homeostatic Model Assessment for Insulin Resistance, LDL-C = low-density lipoprotein cholesterol, OGTT = oral glucose tolerance test, SBP = systolic blood pressure, TMI = tri-ponderal mass index.

### 3.2. Logistic regression analysis of factors associated with glucose metabolism disorder

The univariable analysis indicated that older age, female sex, Tanner stage III or higher (vs stage I), and larger waist circumference were associated with glucose metabolism disorder (Table 3). However, the multivariable analysis did not identify any factors independently associated with glucose metabolism disorder (Table 3).

**Table 3:** Logistic regression analyses of factors associated with glucose metabolism disorder.

	Univariate analysis			Multivariate analysis		
	OR	95% CI	*P* value	OR	95% CI	*P* value
Age	1.137	1.055–1226	.001	1.070	0.963–1.189	.208
Sex						
Male	REF	–	–	REF	–	–
Female	1.645	1.137–2.380	.008	1.385	0.845–2.270	.197
Family history of diabetes	0.920	0.571–1.483	.732			
TMI	0.990	0.919–1.067	.788			
Tanner stage						
I	REF	–	–	REF	–	–
II	1.400	0.878–2.234	.157	1.227	0.752–2.003	.413
III	1.850	1.158–2.955	.010	1.408	0.814–2.437	.221
IV	2.112	1.230–3.625	.007	1.351	0.655–2.787	.416
V	3.877	1.833–8.199	.000	2.207	0.844–5.774	.107
Waist circumference	1.019	1.003–1.035	.019	1.007	0.987–1.027	.512
Total cholesterol	1.154	0.940–1.417	.172			

CI = confidence interval, OR = odds ratio, TMI = tri-ponderal mass index.

### 3.3. Correlations between TMI and indicators related to glucose metabolism disorder

**Figure 1.**
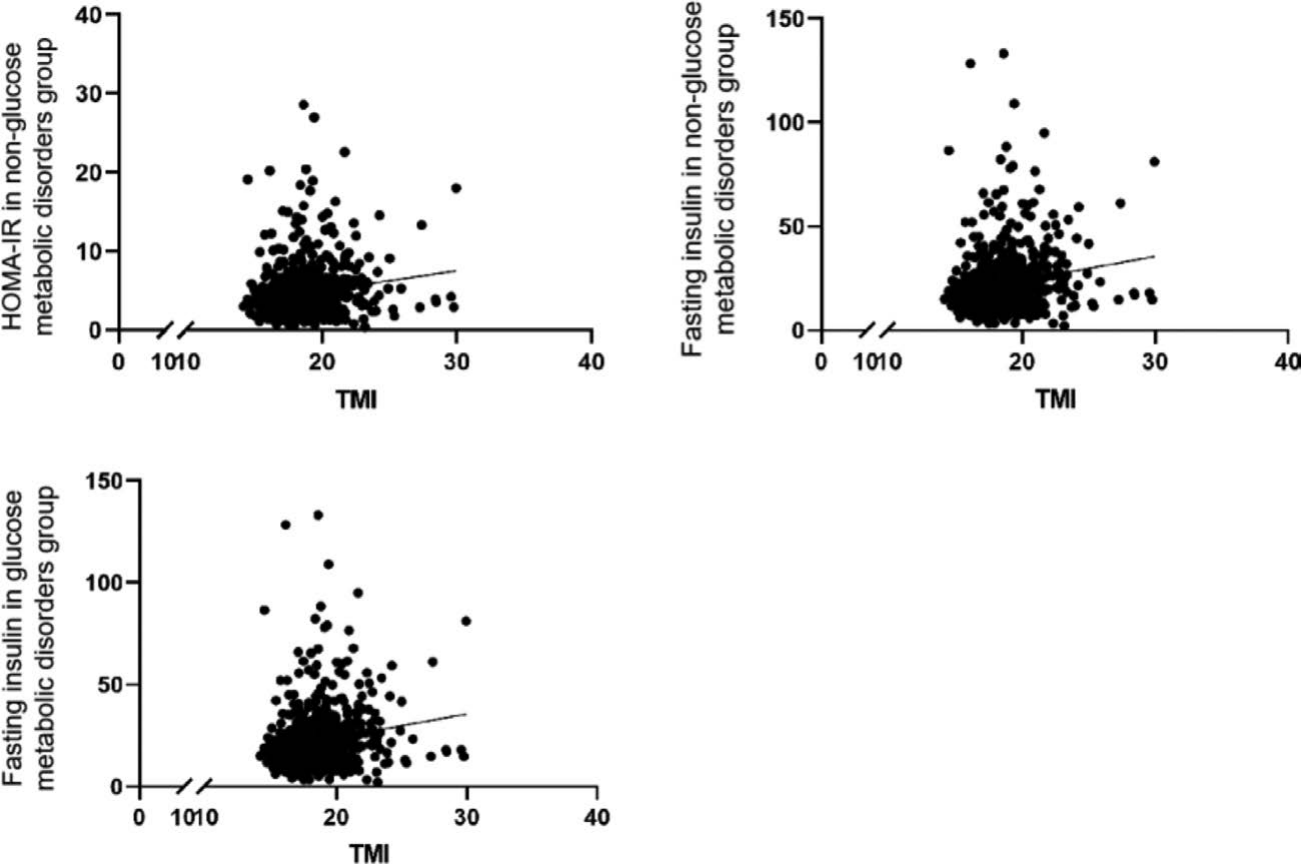
Analyses of the correlations between tri-ponderal mass index (TMI) and indicators of glucose metabolism disorder (fasting blood insulin and Homeostatic Model Assessment for Insulin Resistance [HOMA-IR]). For the analysis, the patients were stratified into a glucose metabolism disorder group and a non-glucose metabolism disorder group.

As summarized in Table 4 and Figure 1, Pearson correlation analysis revealed that TMI was very weakly correlated with FBI (*R* = 0.171, *P* < .001) and HOMA-IR (*R* = 0.168, *P* < .001) in obese patients with glucose metabolism disorder and very weakly correlated with FBI in obese patients without glucose metabolism disorder (*R* = 0.168, *P* = .027). However, TMI was not significantly correlated with FBG, HbA1c, OGTT 2-hour blood glucose level, or OGTT 2-hour blood insulin levels in patients with glucose metabolism disorder or those without (Table 4).

**Table 4:** Pearson correlations between tri-ponderal mass index and indicators related to glucose metabolism disorder.

	Glucose metabolism disorder group		Non-glucose metabolism disorder group	
	R	*P*	R	*P*
TMI vs fasting blood glucose	0.018	.653	0.062	.419
TMI vs fasting blood insulin	0.171	<.001	0.168	.027
TMI vs HOMA-IR	0.168	<.001	0.135	.077
TMI vs HbA1c	0.058	.149	−0.082	.285
TMI vs OGTT 2-h blood glucose	−0.073	.073	0.032	.679
TMI vs OGTT 2-h blood insulin	0.059	.147	0.053	.489

HbA1c = glycated hemoglobin, HOMA-IR = Homeostatic Model Assessment for Insulin Resistance, OGTT = oral glucose tolerance test, TMI = tri-ponderal mass index.

## 4. Discussion

A notable finding of this study is that children with obesity who had glucose metabolism disorder had comparable BMI and TMI to those who did not have glucose metabolism disorder. Furthermore, logistic regression analysis did not identify TMI as a factor associated with glucose metabolism disorder. The associations of TMI with indicators related to glucose metabolism disorder were very weak or negligible. Therefore, TMI may not be a useful indicator to screen for glucose metabolism disorders in children with obesity in China.

Obesity is a known risk factor for T2DM in children.^[6,14]^ In the present study, the prevalence of glucose metabolism disorder in children with obesity in China was 22.0%. Previous studies reported that the prevalence of T2DM in children with obesity was 18.3% in China^[28]^ and 12.6% to 24.6% in other countries.^[29–32]^ Thus, our findings of a high rate of glucose metabolism disorder in children with obesity are broadly in agreement with the results of previous studies.

In this analysis, the patients with glucose metabolism disorder were older, comprised more females, had a higher Tanner index, and had a larger waist circumference than those without glucose metabolism disorder. Female sex has been reported to be a risk factor for metabolic syndrome in children,^[33]^ consistent with our findings. Furthermore, our observations agree with those of prior studies, which concluded that the prevalence of prediabetes in children increased with older age^[34]^ and a larger waist-to-hip ratio.^[35]^

The main finding of this study was that TMI did not differ significantly between children with obesity who had glucose metabolism disorder and those who did not have glucose metabolism disorder. Furthermore, logistic regression analysis did not identify TMI as a factor associated with glucose metabolism disorder. Although previous research has indicated that TMI has no clear advantages over BMI in predicting pediatric glucose metabolism disorder,^[17,36,37]^ several reports concluded that TMI has moderate discriminatory power in detecting metabolic syndrome in children.^[20–22]^ However, these previous studies of TMI as a possible marker of metabolic syndrome did not focus specifically on children with obesity but also included children with normal weight or overweight. It is possible that, although TMI may be predictive of metabolic syndrome in children generally, its predictive value is lost once a threshold level of TMI (i.e., adiposity) is exceeded. Consistent with this suggestion, a study in Iran reported age-dependent optimal cutoff values for TMI of 12.19 to 13.26 kg/m^3^ in boys and 12.19 to 13.26 kg/m^3^ in girls,^[20]^ while an analysis of children in China reported optimal cutoff values for TMI of 14.46 kg/m^3^ in boys and 13.91 kg/m^3^ in girls.^[22]^ The median TMI of the children in the non-glucose metabolism disorder group in the present study was 18.37 kg/m^3^, which is far higher than the optimal cutoff values described in these previous studies. Hence, although TMI might be a useful marker of glucose metabolism disorder in children generally, it may lack clinical utility for the screening of abnormal glucose metabolism in obese children.

This study has some limitations. First, the present analysis only included children with obesity. Therefore, additional research is needed to establish whether TMI might be associated with dysglycemia in the non-obese pediatric population in China. Second, since BMI was affected by age and gender, further analyses are warranted to establish whether the *z*-scores or standard deviation scores for BMI and TMI^[19,20,38]^ might have utility in predicting glucose metabolism disorder in children with obesity in China. Third, it was not determined whether the degree of obesity might influence the utility of TMI in predicting glucose metabolism disorder.

In conclusion, TMI was not associated with glucose metabolism disorder in children with obesity in China. However, given the published data in the literature, further studies are warranted to evaluate the possible role of TMI in the screening of glucose metabolism disorder in children generally, either alone or in combination with other indicators.

## Author contributions

**Conceptualization:** Dongguang Zhang, Yu Yang.

**Data curation:** Dongguang Zhang, Yu Yang, Xian Wu, Li Yang, Qingbo Xu.

**Formal analysis:** Lei Xu, Haiying Zou, Xian Wu, Li Yang, Bin Zhou, Qingbo Xu.

**Writing – original draft:** Dongguang Zhang, Yu Yang, Xian Wu, Li Yang, Qingbo Xu.
